# Skin wound healing improvement in diabetic mice through FTIR microspectroscopy after implanting pluripotent stem cells

**DOI:** 10.1063/5.0130383

**Published:** 2023-02-07

**Authors:** Gustavo J. Vazquez-Zapien, Adriana Martinez-Cuazitl, Alejandra Granados-Jimenez, Miguel Sanchez-Brito, Melissa Guerrero-Ruiz, Alejandro Camacho-Ibarra, Misael A. Miranda-Ruiz, Ian S. Dox-Aguillón, Jesus A. Ramirez-Torres, Monica M. Mata-Miranda

**Affiliations:** 1 Escuela Militar de Medicina, Centro Militar de Ciencias de la Salud, Secretaría de la Defensa Nacional, Mexico City 11200, Mexico; 2 Centro de Investigación y Desarrollo del Ejército y Fuerza Aérea Mexicanos, Secretaría de la Defensa Nacional, Mexico City 11400, Mexico; 3 Escuela Nacional de Medicina y Homeopatía, Instituto Politécnico Nacional, Mexico City 07320, Mexico; 4 Escuela Superior de Cómputo, Instituto Politécnico Nacional, Mexico City 07738, Mexico

## Abstract

Diabetes is a chronic degenerative disease that carries multiple complications. One of the most important complications is the diabetic cutaneous complications, such as skin lesions, ulcerations, and diabetic foot, which are present in 30%–70% of the patients. Currently, the treatments for wound healing include growth factors and cytokines, skin substitutes, hyperbaric oxygen therapy, and skin grafts. However, these treatments are ineffective due to the complex mechanisms involved in developing unhealed wounds. Considering the aforementioned complications, regenerative medicine has focused on this pathology using stem cells to improve these complications. However, it is essential to mention that there is a poor biomolecular understanding of diabetic skin and the effects of treating it with stem cells. For this reason, herein, we investigated the employment of pluripotent stem cells (PSC) in the wound healing process by carrying out morphometric, histological, and Fourier-transform infrared microspectroscopy (FTIRM) analysis. The morphometric analysis was done through a photographic follow-up, measuring the lesion areas. For the histological analysis, hematoxylin & eosin and picrosirius red stains were used to examine the thickness of the epidermis and the cellularity index in the dermis as well as the content and arrangement of collagen type I and III fibers. Finally, for the FTIRM analysis, skin cryosections were obtained and analyzed by employing a Cassegrain objective of 16× of an FTIR microscope coupled to an FTIR spectrometer. For this purpose, 20 mice were divided into two groups according to the treatment they received: the Isotonic Salt Solution (ISS) group and the PSCs group (n = 10). Both groups were induced to diabetes, and six days after diabetes induction, an excisional lesion was made in the dorsal area. Furthermore, using microscopy and FTIRM analysis, the skin healing process on days 7 and 15 post-skin lesion excision was examined. The results showed that the wound healing process over time, considering the lesion size, was similar in both groups; however, the PSCs group evidenced hair follicles in the wound. Moreover, the histological analysis evidenced that the PSCs group exhibited granulation tissue, new vessels, and better polarity of the keratinocytes. In addition, the amount of collagen increased with a good deposition and orientation, highlighting that type III collagen fibers were more abundant in the PSCs. Finally, the FTIR analysis evidenced that the PSCs group exhibited a faster wound healing process. In conclusion, the wounds treated with PSCs showed a more rapid wound healing process, less inflammatory cellular infiltration, and more ordered structures than the ISS group.

## INTRODUCTION

Diabetes is a chronic degenerative disease that occurs when the pancreas does not produce enough insulin or the body cannot effectively use it. In 2019, approximately 463 million adults aged 20 and 79 were living with diabetes; by 2045, this will rise to 700 million.^1^ This illness has become the seventh leading cause of death in the United States and the leading cause of amputation, blindness, and renal failure.^2^ In addition, the cost of wound treatment ranges from 28.1 billion to 96.8 billion US dollars due to the increase in the aging and diabetic population.^3^

According to the American Diabetes Association (ADA), this pathology is a complex chronic disease requiring continuous medical care with multifactorial risk reduction strategies for glycemic control. Moreover, this disease has many medical complications, such as myocardial infarction, cerebrovascular accidents, kidney failure, amputation of lower limbs, and neuropathy.^4^ It has been highlighted that between 30% and 70% of patients with diabetes, both type 1 and type 2, will present a diabetes cutaneous complication at some point during their lifetime, associated with an increased risk of essential outcomes, such as skin lesions, ulcerations, and diabetic foot, which can lead to further significant complications.^5,6^ Furthermore, some studies have demonstrated that most diabetic amputations precede foot ulceration, resulting in severe gangrene or infection.^7^

On the other hand, human skin is a physical barrier with multiple functions; it works as an interface between the human body and its environment, prevents water loss, and protects the body from physical, chemical, and biological insults. Moreover, it contributes to the vascular capacity of the entire body, blood pressure control, and glucose handling.^8^ Nevertheless, some of these functions are disrupted in diabetic people.

As known, wound healing is a physiological reaction to tissue injury involving a complex interplay between numerous cell types, cytokines, mediators, and the vascular system. It begins with homeostasis and ends with scar tissue formation; the necrotic tissue is removed by scavenger cells or separated from living tissue by the process of phagocytosis. The wound healing process consists of four phases: (1) Hemostasis: in this phase, vasoconstriction of blood vessels and platelet aggregation is carried out; the clot provides a matrix for the cells involved in the subsequent steps of hemostasis and inflammation. (2) Inflammation: the goal of the inflammatory phase is to fight against possible bacterial contamination and to activate cytokine secretion; the duration of the inflammatory stage usually lasts several days. (3) Proliferation: this is characterized by granulation (dermis repair), re-epithelialization (formation of a new epidermis), and angiogenesis; this phase can last several weeks. (4) Remodeling: once the fibrin clot is formed, it is replaced by granulation tissue rich in collagen types III and I. In this phase, the wound achieves maximum strength as it matures.^9–11^

It is essential to mention that in diabetes, extracellular matrix proteins undergo glycation-induced modification leading to the formation of advanced glycation end products. This is why collagen is subjected to non-enzymatic glycosylation, provoking some people with diabetes to have thickened, waxy skin.^2,12^

In addition, more than 100 physiological factors are involved in the healing process, and some do not work well in people with diabetes, entailing poor wound healing. These include decreased production of growth factors, alterations in angiogenic response, macrophage function, collagen accumulation, epidermal barrier function, amount of granulation tissue, migration and proliferation of keratinocytes and fibroblasts, number of epidermal nerves, healing bone, and imbalance between the accumulation of the extracellular matrix (ECM) components and their remodeling by matrix metalloproteinases (MMPs).^13^

Current treatments for wound healing include growth factors and cytokines, skin substitutes, hyperbaric oxygen therapy, and skin grafts. However, these conventional treatments are not effective, probably due to the complex mechanisms involved in developing unhealed wounds, which is why regenerative medicine using stem cells could be a novel tool for treating non-healing ulcers.^3^

Therefore, it is essential to analyze the healing process biomolecularly, using precise, fast, and less expensive methods, and to investigate and propose effective treatments that improve healing or increase skin regeneration.

In this sense, Fourier transform infrared (FTIR) spectroscopy is a valuable tool in studying biological samples since it provides information on the molecular structure of organic and inorganic materials. In this technique, the absorption of infrared radiation (IR) occurs when a photon is transferred to a molecule and excites it to a higher energy state, giving rise to vibrations of molecular bonds, which occur at different wave sizes or frequencies in the IR region of the light spectrum.^14^

Different biomolecules, such as lipids, proteins, carbohydrates, and nucleic acids, can be detected in biological materials with specific chemical structures. In addition, by coupling FTIR spectroscopy with optical microscopy, analysis using FTIR microspectroscopy (FTIRM) has been possible, which allows the visualization and mapping of functional groups and molecular arrangements.^15^ This method has been called “chemical photography,” as semi-quantitative information can be obtained from IR spectra using standard image processing software.^16^

The FTIRM is sensitive to determining the distribution and orientation of various components, which has been used to recognize multiple pathologies; this technique makes it possible to detect concentrations of molecules using the area under the curve of specific peaks in the spectrum as well as the radii between particular areas.^15^ For example, it is possible to analyze the secondary structure of proteins employing the second derivative or deconvolution, examining bands corresponding to α-helices, β sheets, β turns, and disordered structure.^17^ The specificity and advantages of using the second derivative have made it possible to determine the number and position of peaks of interest, especially in the region of amide I proteins,^18^ and hence, its usefulness in wound regeneration could reveal molecular data not described far.

In this sense, regenerative medicine has focused on diabetes, not only in the search to decrease glucose levels but also in the pursuit to improve the healing process due to the increased risk of developing skin lesions in diabetic people. Therefore, due to the poor biomolecular understanding of diabetic skin and the effects of treating it with stem cells, in this work, we analyzed in a nondestructive and specific way an experimental treatment of PSCs in a post-skin lesion excision diabetic murine model, correlating the morphometric, histological, and microspectroscopic characteristics of the wound.

## RESULTS

### Murine diabetic model

**FIG. 1.**
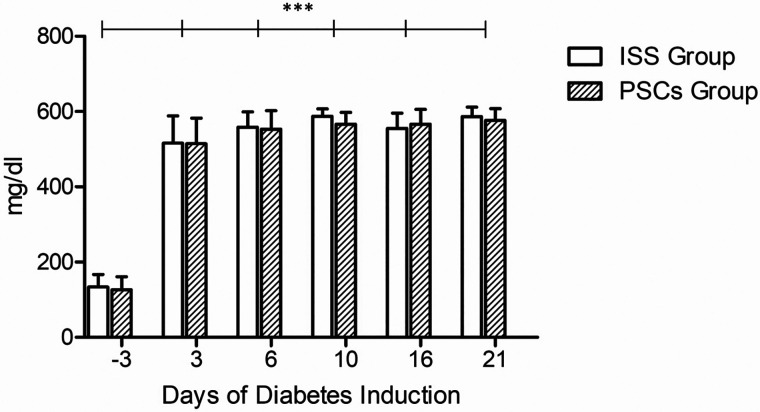
Capillary glucose. Three days after diabetes induction, both groups showed higher glucose levels. The data shown are mean ± SD (error bars), ^***^p <0.001.

As previously mentioned, the capillary blood glucose was monitored. Before diabetes induction, capillary glucose was evaluated in both groups. The ISS group exhibited 133.70 ± 31.68 mg/dl, and the PSCs group exhibited 126.60 ± 32.37 mg/dl. Three days after diabetes induction, both groups showed higher glucose levels; the ISS group presented 515.40 ± 68.97 mg/dl, and the PSCs group presented 516.00 ± 63.06 mg/dl; no statistical significance was observed between the groups along the skin healing process. On day 21, the ISS group presented 586.40 ± 24.12 mg/dl, and the PSCs group presented 576.30 ± 29.44 mg/dl (Fig. 1), highlighting that no treatment was given for the diabetes control.

### Morphometric analysis of the excisional lesion

**FIG. 2.**
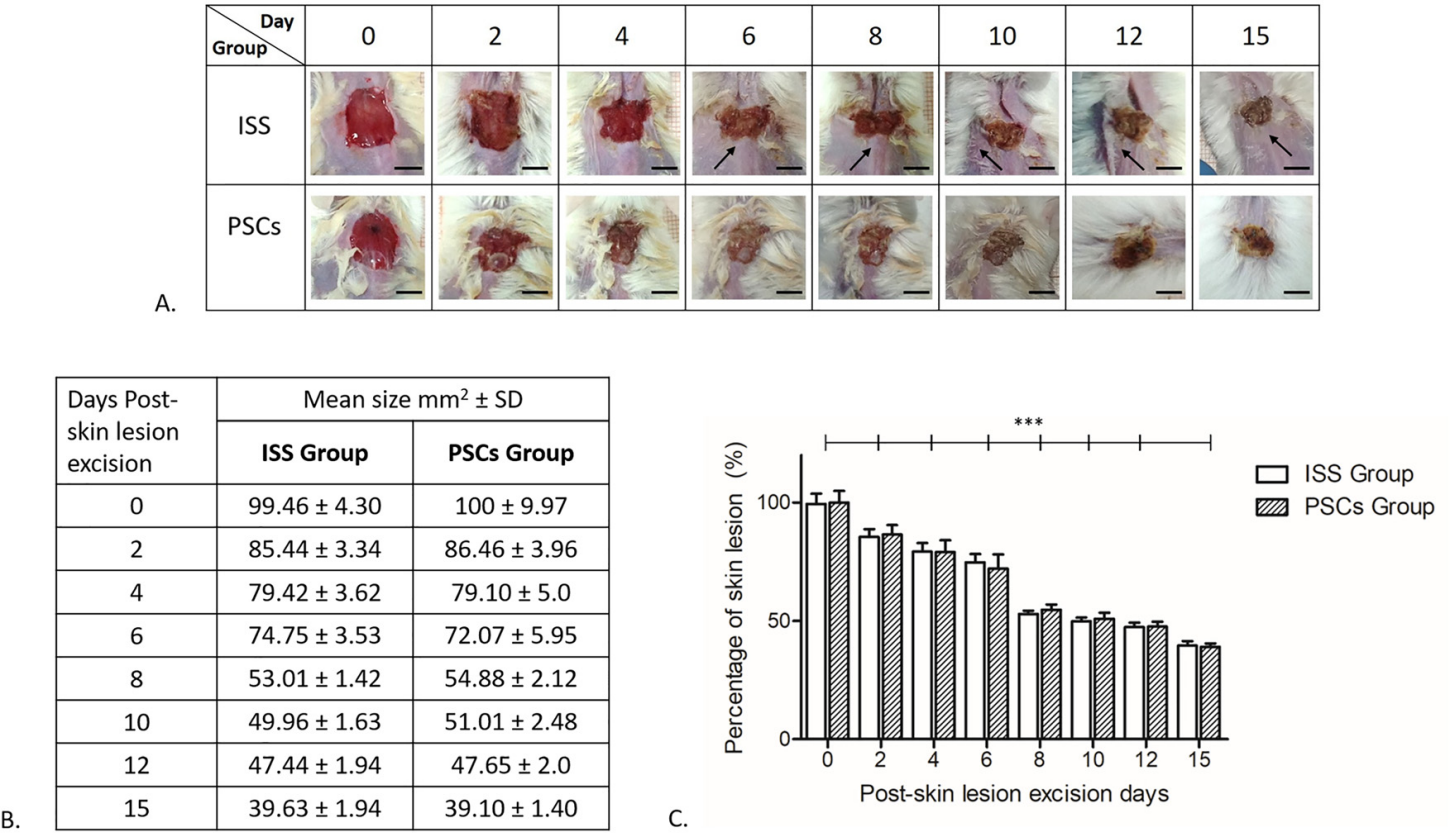
Morphometric analysis. (a) Representative photos of one mouse of each group, the wound healing process is shown, indicating that no hair surrounding the wound was observed in the ISS group (**↑**). Scale bar represents 5 mm. (b) Table of means of the lesion areas, both groups (ISS and PSCs) showed at day 15 a lesion mean size of 39 mm^2^, showing no statistical significance between groups at any day. (c) Percentage of skin lesions over time; statistical significance of how the percentage of the skin lesion diminished is shown; however, no statistical difference was reported between groups. The data shown are mean ± SD (error bars), ^***^p <0.001.

Morphological analysis was performed by a photograph record obtained when the injury was made (day 0) and then on days 2, 4, 6, 8, 10, 12, and 15. In Fig. 2(a), representative photos of one mouse of each group are shown; the wound healing process over time can be noticed, highlighting that in the ISS group, no hair is evidenced, which is in contrast with the PSCs group where the hair surrounds the wound; even more, some hairs are shown in the scab.

In Fig. 2(b), the means of the lesion areas are tabulated. On day 0, it can be observed that the skin lesion excisions in both groups are pretty similar; the ISS group presented an area lesion of 99.46 ± 4.07 mm^2^, and the PSCs group presented an area lesion of 99.99 ± 4.71 mm^2^. Two days post-skin lesion excision in both groups, the area lesion was reduced; the ISS presented an area lesion of 85.44 ± 3.17 mm^2^, and the PSCs group presented an area lesion of 86.46 ± 3.76 mm^2^. Both groups showed a decrease in the lesion area throughout the healing time. However, no statistical significance was observed between the groups. On day 15 post-skin lesion excision, the ISS group presented an area lesion of 39.62 ± 1.73 mm^2^ and the PSCs group presented an area lesion of 39.10 ± 1.25 mm^2^. Figure 2(c) shows the percentage of skin lesions over time.

### Histological analysis

**FIG. 3.**
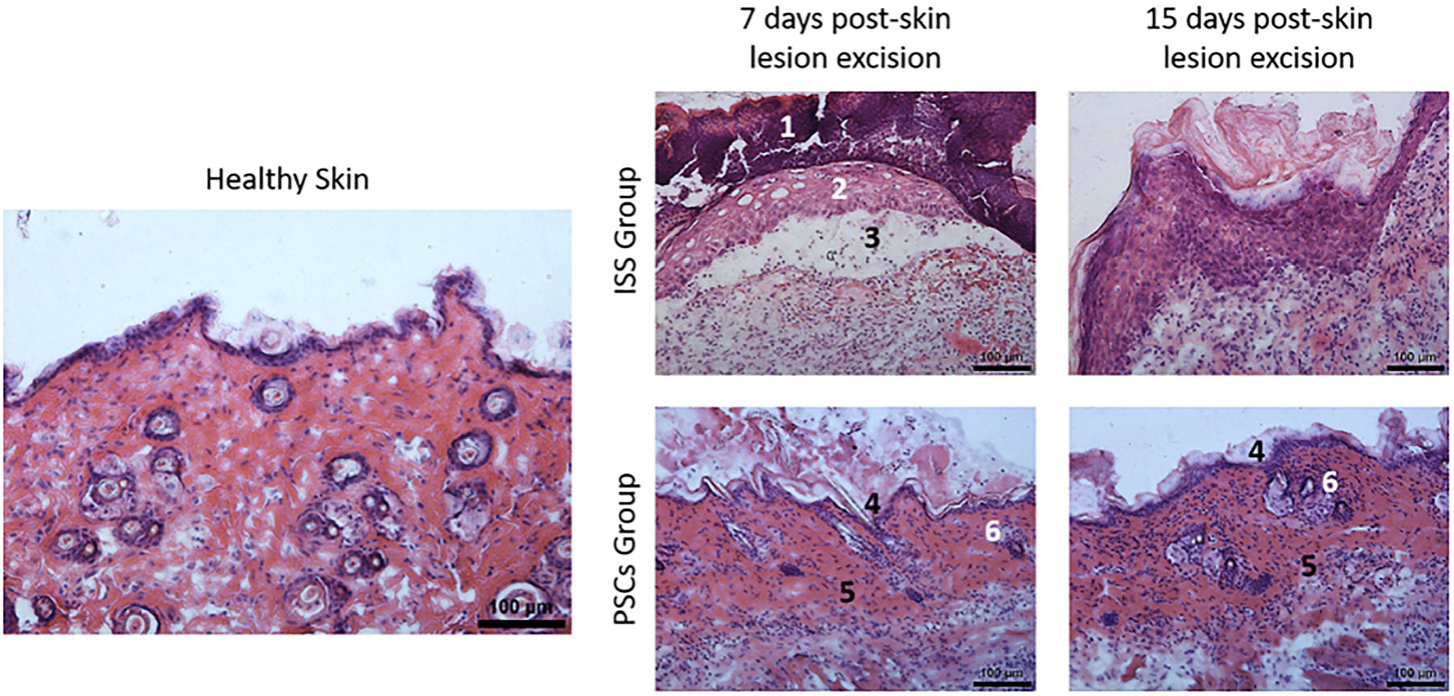
Histological analysis. Representative hematoxylin and eosin-stained histopathological samples corresponding to skin samples of ISS and PSCs groups at days 7 and 15 post-skin lesion excision (100×). On day 7, post-skin lesion excision in the ISS group, a hematic scab (1) and subcostraceous epithelialization (2) were observed. Moreover, a separation of the papillary dermis (3) due to edema was also identified as great cellularity, highlighting the lack of skin annexes. In contrast, in the PSCs group, the scab was less solid, and there was no evidence of the separation of the papillary dermis (4) observed in the ISS, and skin annexes were observed. On day 15 post-skin lesion excision, a greater amount of granulation tissue was evidenced in the PSCS group (5), highlighting the presence of hair follicles (6).

The histopathological samples of both groups (ISS and PSCs) were stained employing hematoxylin & eosin and were evaluated at 7 and 15 days post-skin lesion excision. In Fig. 3, representative microphotographs are shown; along the wound healing process, different stages were identified, including coagulation, granulation tissue formation, re-epithelialization, and ECM remodeling. On day 7 post-skin lesion excision in the ISS group, a hematic scab (1) that lines the wound bed can be evidenced and the subcostraceous epithelialization (2) caused by proteolytic enzymes that make their way under the fibrin crust (from which they can get fed). In addition, a separation of the papillary dermis (3) due to edema and migration of inflammatory cells was observed, and finally, the lack of skin annexes (hair follicles, sebaceous, and sweat glands) was observed.

In contrast, in the PSCs group, the scab is less solid, and the subcostraceous epithelialization process (4) is not as evidenced as in the ISS group, highlighting the separation of the papillary dermis was observed in the ISS was not observed in the PSCs group. Moreover, the collagen fibers in the dermis were examined, noticing a higher collagen fiber density in the PSCs group than in the ISS (these are observed in pink). Furthermore, it is essential to mention that, as seen in the macroscopic examination, skin annexes were observed in the dermis of the PSCs group.

On day 15, post-skin lesion excision, in the macroscopic analysis through the photograph record, it can be observed that the scab remains; nevertheless, due to the handling of the sample, it was lost in the process of the histological technic. However, an epithelialization below the covering surface where the scab should have been placed was observed; it was also evidenced that the epidermis thickened with a more significant number of keratinocytes, persisting a slight sign of edema in the dermis layer. However, a demarcation line comprised mainly of polymorphonuclear leukocytes (PMN) and granulation tissue (5) is observed. When comparing the healing process in the ISS group on day 7 post-skin lesion excision, the better polarity of the keratinocytes and a greater density of collagen fibers were observed. In contrast, the PSCs group on day 15 exhibited a greater amount of granulation tissue (5), as well as collagen fibers, highlighting the presence of skin annexes [hair follicles (6)] (Fig. 3).

**FIG. 4.**
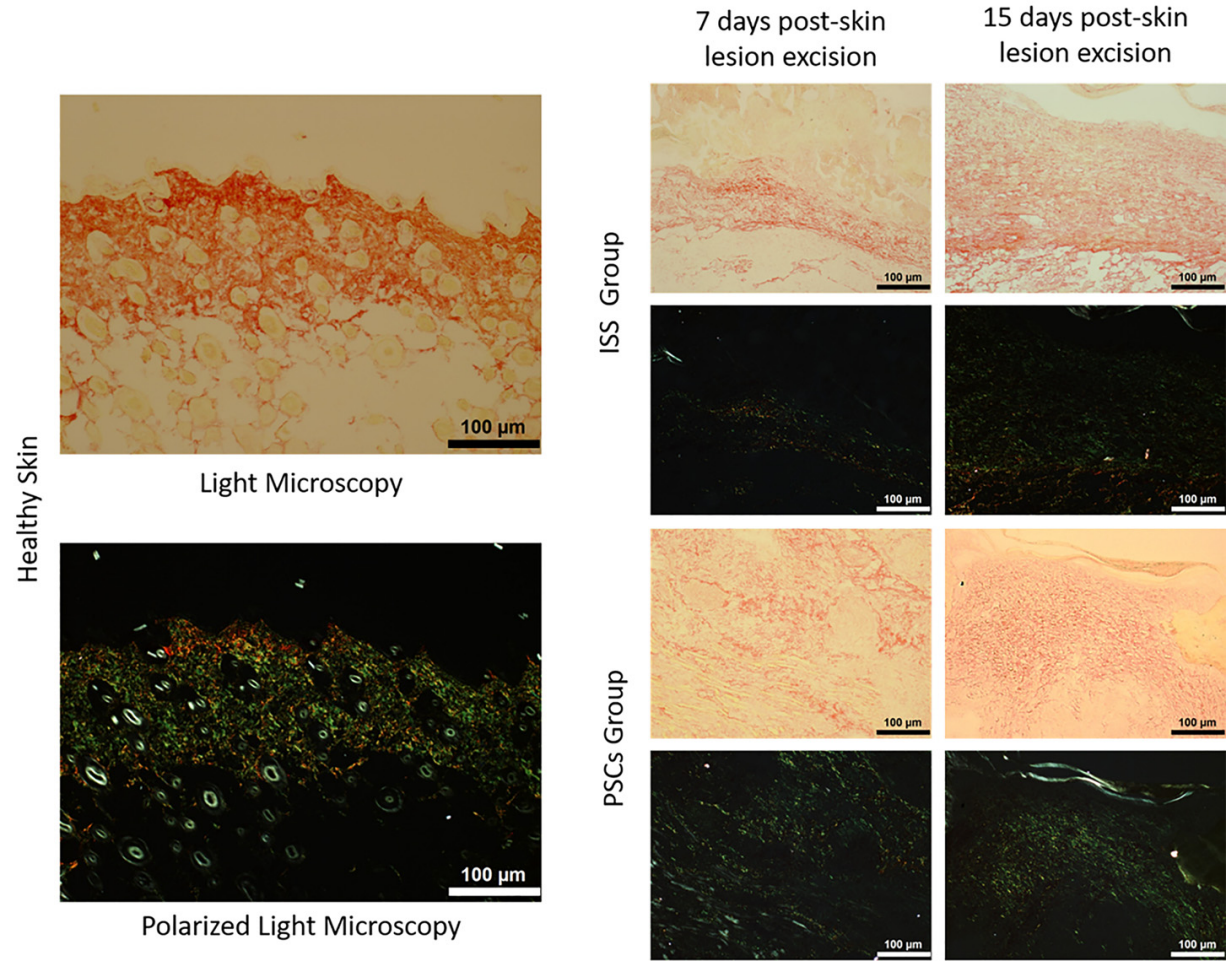
Picrosirius red stain (100×). Type I collagen fibers are seen in yellow-orange, and type III collagen fibers in green. The type III collagen fibers are more abundant in the PSCs group than in the ISS group.

Figure 4 shows the histopathological analysis at 7 and 15 days post-skin lesion excision of both groups (ISS and PSCs) through picrosirius red stain. The use of light microscopy allows the distinction of collagen fibers, which are colored in red. On the other hand, polarized light microscopy produces a birefringence, provoking that type I collagen fibers (thick fibers) to be seen in yellow-orange and type III collagen fibers (thin fibers) to be seen in green. Therefore, the emitted luminescence was evaluated to determine the content of the collagen fibers. As seen, type I collagen fibers are abundant in healthy skin (171 141.34 ± 27.43 lm), but they considerably decreased at day 7 post-skin lesion excision in the ISS (42 683.7 ± 26.87 lm) and in the PSCs groups (115 126.55 ± 14.92 lm).

**FIG. 5.**
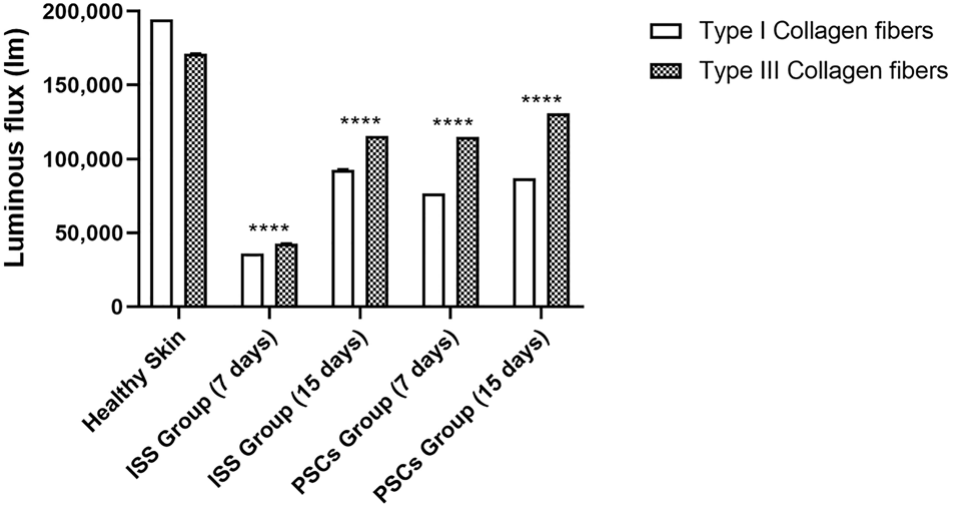
Luminescence quantification emitted by the type I and III collagen fibers. Type I and III collagen fibers considerably decreased at day 7 post-skin lesion excision in both groups, showing a partial recovery at day 15 post-skin lesion excision. The content of type I and III collagen fibers was greater in the PSCs group at days 7 and 15 post-skin lesion excision. The data shown are mean ± SD (error bars), ^****^p <0.0001.

Moreover, at day 15 post-skin lesion excision, these fibers showed an increment in the ISS group (115 719.11 ± 16.4 lm) and PSCs group (130 955 ± 15.54 lm). In the same way, the type III collagen fibers were abundant in the healthy skin (194 439.25 ± 30.02 lm) and decreased at day 7 post-skin lesion excision in the ISS group (36 123.76 ± 27.87 lm) and the PSCs group (76 745.96 ± 14.25 lm); the experimental groups showed a higher content of type III collagen fibers, but they slightly recovered at day 15 post-skin lesion excision in the ISS group (92 989.32 ± 17.5 lm) and also in the PSCs groups (87 111.29 ± 11.67 lm). However, the type III collagen fibers are more abundant in the PSCs group compared to the ISS group (Fig. 5).

**FIG. 6.**
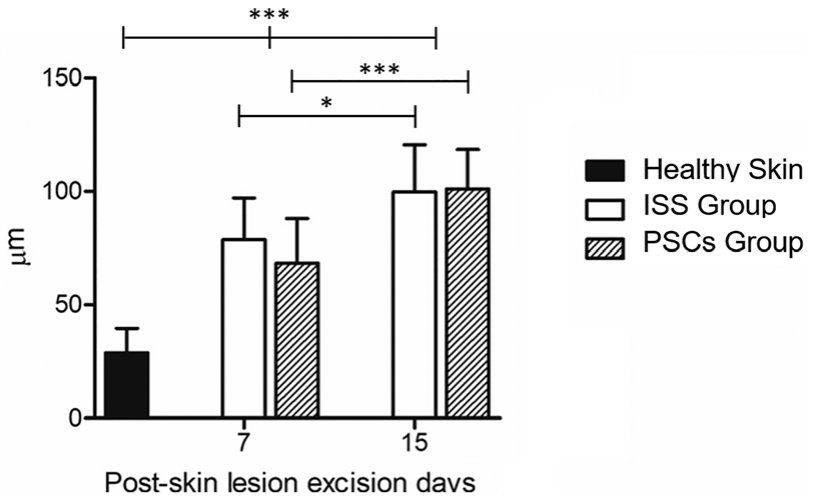
Skin thickness. Both groups (ISS and PSCs groups) showed increased epidermis thickness on days 7 and 15 post-skin lesion excision. However, no statistical significance between groups was observed at any time. The data shown are the mean ± SD (error bars), ^*^p <0.05, ^***^p <0.001.

The epidermis thickness was also evaluated; Fig. 6 depicted the epidermis thickness of healthy mice skin and scarred treated with ISS or PSCs, according to their group. As observed, the thickness of the epidermis increased throughout the healing time. For example, the thickness of a healthy epidermis was 28.87 ± 10.81 *μ*m; nevertheless, the epidermis of the ISS group healed on days 7 and 15 post-skin lesion excision measured 78.63 ± 18.55 and 99.87 ± 20.84 *μ*m, respectively. In the same way, the epidermis of the PSCs group healed at 7 and 15 days post-skin lesion excision also showed an increment in the epidermis thickness, measuring 68.38 ± 19.69 and 101.2 ± 17.48 *μ*m, respectively, observing that even though no statistical significance was found, the ISS group presented a thicker epidermis that the PSCs group at day 7 post-skin lesion excision. Nevertheless, on day 15, the PSCs group exhibited a thicker epidermis than the ISS group.

**FIG. 7.**
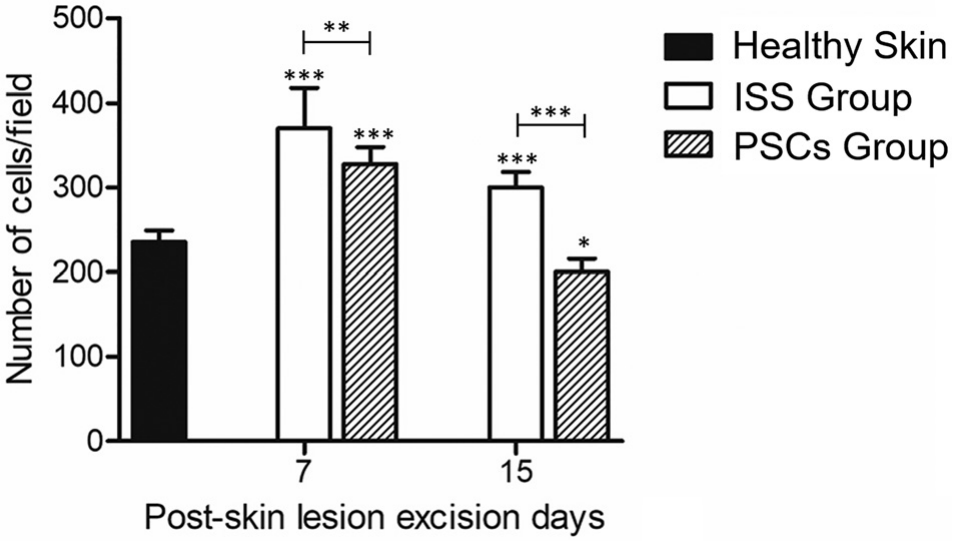
Cellularity. The cellularity increased on day 7 post-skin lesion excision in both groups, diminishing on day 15 post-skin lesion excision. The PSCs group showed less cellularity than the ISS group at days 7 and 15 post-skin lesion excision, evidencing statistical significance. The data shown are the mean ± SD (error bars), ^*^p <0.05, ^**^p <0.01, ^***^p <0.001.

As previously mentioned, cellularity was also evaluated, for which purpose the cellularity of healthy mice skin dermis was analyzed and compared to scarred dermis treated with ISS or PSCs at days 7 and 15 post-skin lesion excision. Figure 7 shows that both groups' cellularity increased at day 7 post-skin lesion excision. However, the PSCs group showed less cellularity than the ISS group. Moreover, on day 15 post-skin lesion excision, the cellularity diminished, but it remained increased compared to healthy skin in the ISS group. Nonetheless, the PSCs group exhibited less cellularity than the healthy skin, showing statistical significance.

### FTIR microspectroscopy

**FIG. 8.**
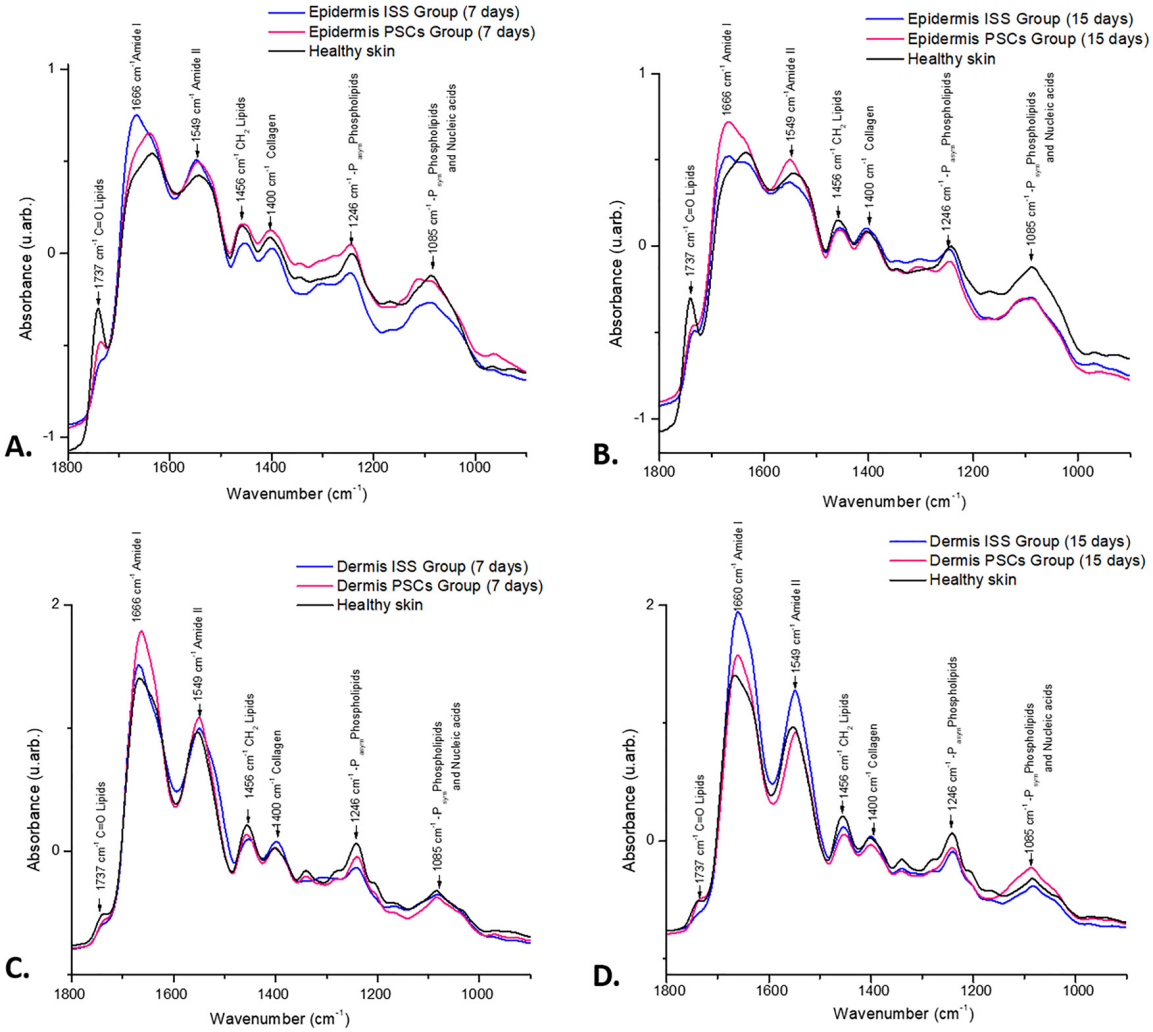
FTIR microspectroscopy (FTIRM) spectra. (a) FTIRM spectra of the epidermis at day 7 post-skin lesion excision in the ISS and PSCs groups. It can be observed that the bands related to lipids, collagen, phospholipids, and nuclei acids showed a higher absorbance in the PSCs group. (b) FTIRM spectra of the epidermis at day 15 post-skin lesion excision in the ISS and PSCs groups. It is shown that the bands related to amide I and amide II had a higher absorbance in the PSCs group. (c) FTIRM spectra of the dermis at day 7 post-skin lesion excision in the ISS and PSCs groups. It is evidenced that the bands related to amide I, amide II, lipids, and phospholipids depicted a higher absorbance in the PSCs group. (d) FTIRM spectra of the dermis at day 15 post-skin lesion excision in the ISS and PSCs groups. It is shown that the bands related to amide I, amide II, lipids, and collagen had a higher absorbance in the ISS group.

Regarding the spectroscopic results using FTIRM, Fig. 8 shows the normalized and averaged FTIR spectra of the epidermis and dermis of healthy skin, as well as skin biopsies obtained on days 7 and 15 post-skin lesion excision of ISS and PSCs groups, where the absorption bands related to lipids (1737 and 1456 cm^−1^), proteins [amide I (1666 cm^−1^) and amide II (1549 cm^−1^)], collagen (1400 cm^−1^), phospholipids (1246 cm^−1^), and nucleic acids (1085 cm^−1^) are depicted.

It can be observed that the band associated with lipids showed a higher absorbance in the PSCs group compared to the ISS group at the different analyzed times. Moreover, the bands related to amides I and II exhibited a higher absorbance in the dermis section of both groups.

Furthermore, the band associated with phospholipids showed a higher absorbance in the epidermis and dermis sections on day 7 than the ISS group; nevertheless, on day 15 in the epidermis sections, the phospholipids band was higher in the ISS group. However, this band remains a higher absorbance in the PSCs group in the dermis section. Finally, the band related to nucleic acids showed a higher absorbance in the epidermis section of the PSCs group on day 7; however, on day 15, the ISS and the PSCs group presented similar absorbance. Moreover, in the dermis section, the nucleic acid band exhibited a slight increase in absorbance in the ISS group; nevertheless, on day 15, the PSCs group presented a higher absorbance.

**FIG. 9.**
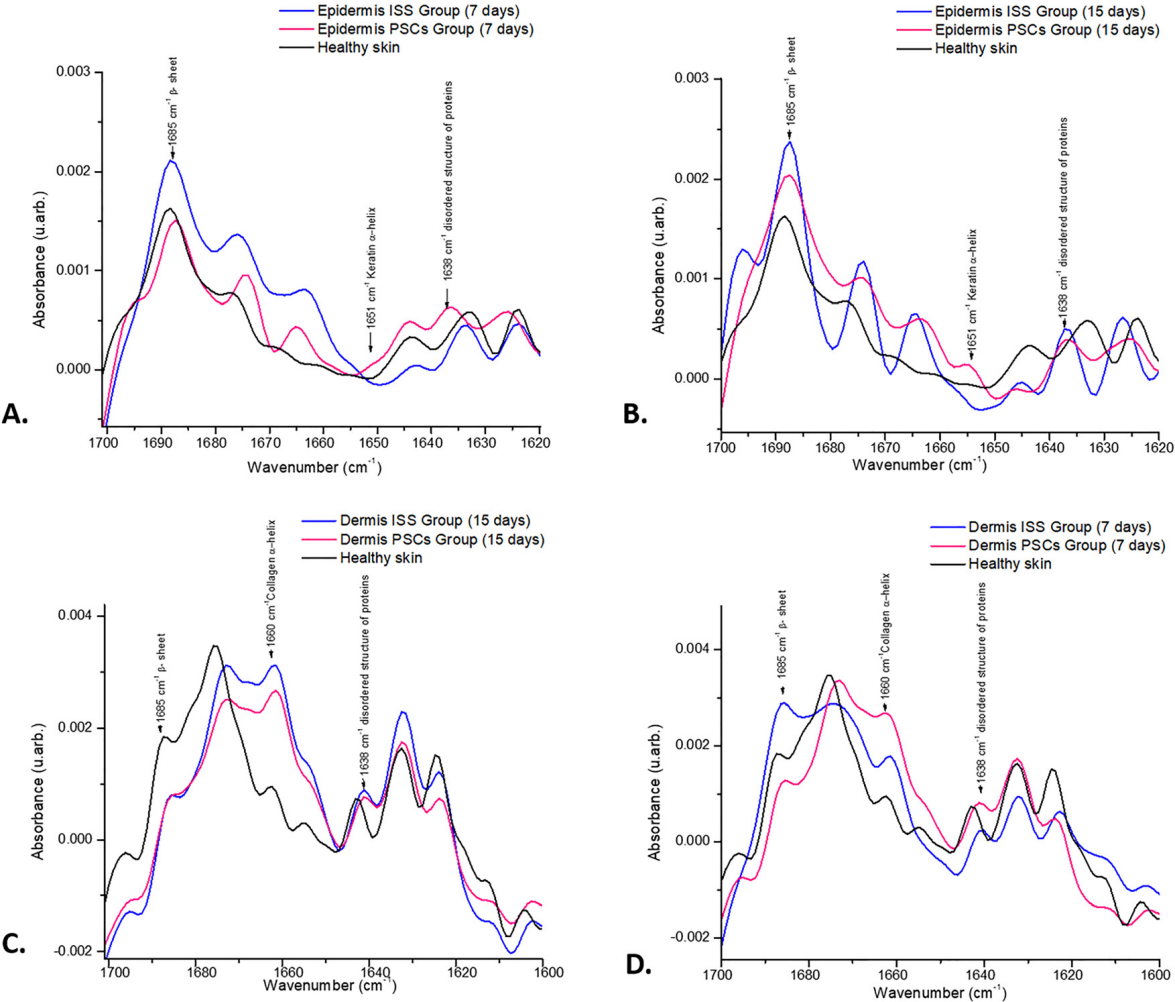
Second derivative of the normalized FTIRM spectra. (a) Second derivative FTIRM spectra of epidermis at day 7 post-skin lesion excision in the ISS and PSCs groups. It is observed that the ISS group showed a higher absorbance than the PSCs group in the band associated with β sheet; in contrary, the bands related to keratin α-helices and disordered structure of proteins evidenced higher absorbance in the PSCs group than the ISS group. (b) Second derivative FTIRM spectra of epidermis at day 15 post-skin lesion excision in ISS and PSCs groups. The ISS group evidenced a higher absorbance than the PSCs group in the bands related to β sheet and disordered structure of proteins; however, the band associated with keratin α-helices depicted a higher absorbance in the PSCs group. (c) Second derivative FTIRM spectra of the dermis at day 7 post-skin lesion excision in the ISS and PSCs groups. It is evidenced that the bands related to collagen α-helices and the disordered structure of proteins expressed a higher absorbance in the PSCs group than in the ISS group. In contrary, the band associated with the β sheet showed a higher absorbance in the ISS group than in the PSCs group. (d) Second derivative FTIRM spectra of the dermis at day 15 post-skin lesion excision in the ISS and PSCs groups. The bands related to collagen α-helices and the disordered structure of proteins expressed a higher absorbance in the ISS group than in the PSCs group.

On the other hand, to specifically analyze the secondary structure of proteins, the second derivative of normalized FTIR spectra of the epidermis and dermis of healthy skin as well as skin biopsies obtained on days 7 and 15 post-skin lesion excision of ISS and PSCs groups in the amide I region of proteins (1700–1600 cm^−1^) was calculated (Fig. 9), where the following bands are observed: β sheets at 1685 cm^−1^, α-helices at 1651 cm^−1^ corresponding to the keratin of the epidermis, and at 1660 cm^−1^ to collagen dermis. Moreover, the disordered structure of proteins at 1638 cm^−1^ was also detected. In the epidermis sections, it can be observed that the band attributed to β sheets showed a higher absorbance in the ISS groups on days 7 and 15; in contrast, the band associated with keratin exhibited a higher absorbance in the PSCs group on days 7 and 15. Finally, in the epidermis section, the band related to the disordered structure of proteins showed a higher absorbance in the PSCs group; nevertheless, on day 15, the ISS group exhibited a higher absorbance. In the same way, in the dermis sections, the band attributed to β sheets at 1685 cm^−1^ showed a higher absorbance in the ISS group on day 7; nevertheless, on day 15, the absorbance decreased. The band associated with collagen at 1660 cm^−1^ showed a higher absorbance in the PSCs group on day 7; however, on day 15, the ISS group presented a higher absorbance. Regarding the disordered structure of the proteins at 1638 cm^−1^, this band showed a higher absorbance in the PSCs group at day 7 in the epidermis and dermis section than in the ISS group. Nevertheless, in both sections, this band evidenced a higher absorbance in the ISS group than in the PSCs group on day 15.

### Mapping analysis

**FIG. 10.**
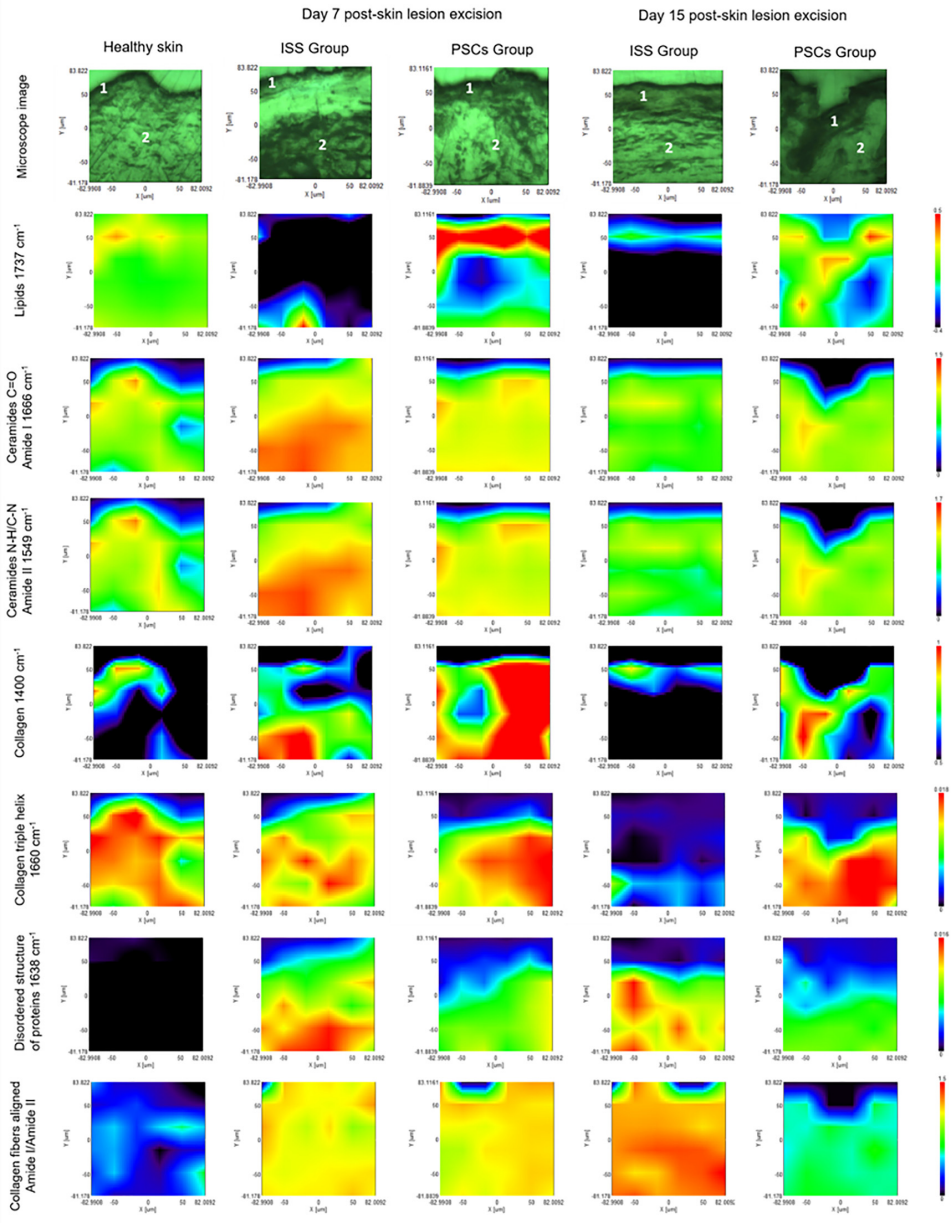
Micro-FTIR images (FTIRI) of the skin samples of the ISS and PSCs groups at 7 and 15 days post-skin lesion excision, showing the biochemical map of different biomolecules and their biodistribution. Epidermis (1) and dermis (2) can be distinguished. The lipids analysis (1737 cm^−1^) demonstrated a higher amount of this molecule in the PSCs group than in the ISS group. In contrary, the ceramides of amide I (1666 cm^−1^) and ceramides of amide II (1549 cm^−1^) analysis showed a great amount of these biomolecules in the ISS group at day 7, highlighting that in the collagen (1400 cm^−1^) and collagen triple helix (1660 cm^−1^) analysis the concentration of these molecules diminished over time in the ISS group. However, these molecules showed a greater amount in the PSCs group than in the ISS group. Finally, the analysis of the disordered structure of proteins (1638 cm^−1^) and collagen fibers aligned (amide I/amide II) showed that the ISS group evidenced a major content. Red and blue colors represent the strong and weak absorption of the infrared beam.

The histological localization and concentration of some crucial molecules in skin bioconformation, including lipids, ceramides C=O amide I, ceramides N–H/C–N amide II, collagen, collagen triple helix, and β-sheet structure content was developed using the IQ mapping function. Figure 10 shows a representative image of the mapping analysis. The microscope image illustrates the skin biopsy in which the analysis was carried out; in the same way, the total absorbance images from FTIRI are presented. Each image represents the integrated absorbance of a specific band of the IR spectra for each pixel of the MCT detector; the red and blue colors represent the strong and weak absorption of the infrared beam.

According to analysis mentioned above, the PSCs group on days 7 and 15 post-skin lesion excision showed a higher lipid content than the ISS group. Nevertheless, ceramides of amide I and amide II presented a greater content in the ISS group on day 7, which significantly decreased on day 15; however, in the PSCs group, the ceramide content remained expressed in a very subtle way. The analysis of the collagen content showed that this molecule showed a higher absorbance on day 7 in both groups, but on day 15, this molecule decreased considerably in the ISS group. The collagen fibers aligned were analyzed through the amide I/amide II ratio, whereas the previously mentioned higher values represent an unordered collagen fiber structure with multiple directions; on day 7, both groups showed almost the same organization of collagen fibers. Nevertheless, on day 15, it can be noticed that the ISS group exhibited an unordered collagen fiber structure, which is in contrast with the PSCs group, which showed ordered fibers. Furthermore, the collagen triple helix analysis evidenced that the PSCs group on days 7 and 15 showed a significant content of this molecule; as seen in the collagen content, the collagen triple helix in the ISS group decreased considerably on day 15. Finally, the disordered structure of proteins and collagen fibers aligned exhibited a great content in the ISS group on days 7 and 15 post-skin lesion excision compared to the PSC group. It is important to mention that all the mapping analysis of the PSCs group at day 15 were quite similar to those presented in healthy skin.

### Biochemical content

**FIG. 11.**
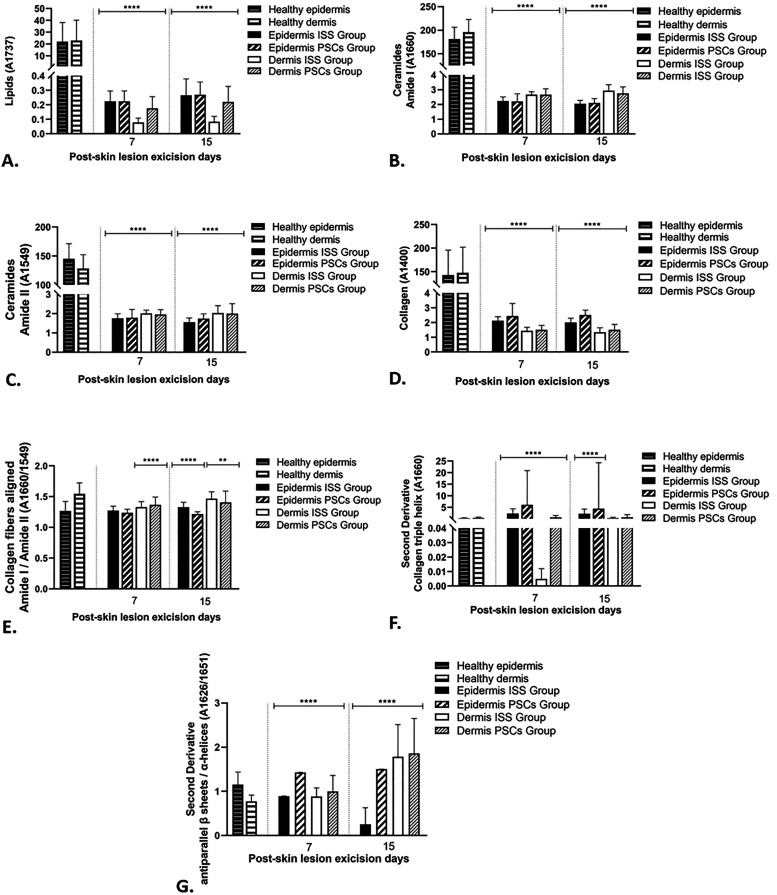
Biochemical content of (a) lipids (1737 cm^−1^), the content of lipids slightly increased at day 15 post-skin lesion excision in the epidermis and dermis sections in both groups, but it retained a low content in the dermis of the ISS group at day 15 post-skin lesion excision compared to the PSCs group. (b) Ceramides of amide I (1666 cm^−1^) and (c) ceramides of amide II (1549 cm^−1^), the content of the ceramides was greater in the dermis than in the epidermis of both groups at days 7 and 15 post-skin lesion excision. (d) Collagen (1400 cm^−1^), the collagen content was higher in the epidermis of both groups at days 7 and 15 post-skin lesion excision compared to the dermis section. However, the PSCs group showed a greater content of this molecule in the epidermis and dermis sections at days 7 and 15 post-skin lesion excision than the ISS group. (e) Collagen fibers aligned (amide I/amide II), this correlation evidenced a greater amount in the dermis of the ISS and PSCs groups at days 7 and 15 post-skin lesion excision compared to the epidermis section. However, the ISS group showed a greater content of this correlation in the epidermis and dermis sections at days 7 and 15 post-skin lesion excision than the PSCs group. (f) Collagen triple helix (1660 cm^−1^), the molecule's content was near to the ground; however, the epidermis sections of the PSCs group at days 7 and 15 post-skin lesion excision showed a major content than the ISS group. (g) α–β transition. It is observed how this relation increased at day 15 post-skin lesion excision in the dermis section of both groups, retaining a greater content in the PSCs group compared to the ISS group. The content of the molecules and relations attributed to the collagen fibers aligned and α–β transition were calculated through the areas under the curve of the bands related to the analyzed molecules. The data shown are mean ± SD (error bars), ^**^p <0.01, ^***^p <0.001, and ^****^p <0.0001.

The areas under the curve of the bands related to lipids, ceramides C=O amide I, ceramides N–H/C–N amide II, collagen, collagen fibers aligned, collagen triple helix, and *α*–*β* transition were calculated in both groups on days 7 and 15 post-skin lesion excision as well as in healthy skin (Fig. 11). It is noticed that lipids, ceramides, and collagen decreased in both groups (ISS and PSCs) with respect to the healthy skin. Regarding lipid content, this molecule increased over time; moreover, the PSCs group in the dermis section showed a more significant amount than the ISS group on days 7 and 15 post-skin lesion excision; nevertheless, the epidermis section did not show differences in content. About the content of ceramide C=O amide I and ceramides N–H/C–N amide II, these molecules decreased in the epidermis section on day 15 post-skin lesion excision compared to day 7. In contrast, it slightly increased on day 15 in the dermis section; however, it is essential to mention that the dermis section presented greater biomolecule content. Regarding collagen amount, its content was more significant in the epidermis sections on days 7 and 15 post-skin lesion excision, but when compared between groups, as seen in the mapping analysis, the PSCs group showed more collagen in the epidermis and dermis sections on day 7 and 15. About the collagen fibers aligned, analyzed with the amide I/amide II ratio, no statistical differences were evidenced on day 7 between groups; nevertheless, on day 15, the ISS group showed a more significant amount of this biomolecule. The analysis of the collagen triple helix showed that the epidermis region has more content of this molecule at days 7 and 15 post-skin lesion excision than the ISS group. Finally, the content of α–β transition was higher in the PSCs group than in the ISS group on days 7 and 15 post-skin lesion excision in the epidermis and dermis sections, highlighting that a decrement was observed on day 15 in the epidermis section of the ISS group. Moreover, on day 15 in the dermis section, an increasement was evidenced.

## DISCUSSION

This experimental study was designed to examine the impact of PSCs as a treatment in the cicatrization process in a diabetes skin lesion, for the purpose of which an excisional skin model in diabetic mice was developed. The lesions were treated with PSCs and ISS according to the group that the animals belonged to, and different characteristics of the wound, such as morphometric, histological, and microspectroscopic characteristics, were studied on days 7 and 15 post-skin lesion excision.

In this research, we decided to use male mice as it has been reported that the male skin is 40% stronger due to a much thicker dermis, while female skin exhibits a thicker epidermis.^21^ Moreover, some studies have concluded that in females mice, estrogens such as 17β-estradiol accelerate the wound healing process.^22^

For the development of diabetic mice in this research, we used STZ, which many authors have used to induce diabetes in animal models. King has stated that STZ is one of the leading compounds to chemically cause models of type 1 diabetes, provoking a high percentage of destruction of endogenous beta cells. Moreover, after i.p. administration, it enters the pancreatic beta cell through the Glut-2 transporter, provoking the alkylation of the DNA and leading to hyperglycemia. King has declared that mice's ideal single high dose ranges from 100 to 200 mg/kg.^23^ The dose employed in this research was 175 mg/kg, with which stable high glycemic values around 500 mg/dl were reached, and it remained high until the end of the experiment, 586.40 ± 24.12 mg/dl for the ISS group and 576.30 ± 29.44 mg/dl for the PSCs group.

Regarding the excisional lesion model, Ma *et al.* investigated the therapeutic effects of mesenchymal stem cells (MSCs) and secretome in the excisional wound model, making a full-thickness skin excision of 2.5 × 2.5 cm^2^ on the dorsal area of adult female SD rats.^3^ Nguyen *et al.* evaluated the topical mineralocorticoid receptor blockade in cutaneous wound healing in diabetic mice using female mice, in which they generated a 6-mm biopsy punch.^7^ Moreover, considering that the wound size should be proportional to the animal size and that female mouse skin exhibits a thicker epidermis and subcutaneous layer,^21^ in this research, we made an excision of 1 × 1 cm^2^ on the dorsal area of adult male diabetic mice.

About the wound healing process, unlike Ma *et al.*, who reported that the wound healing process was completed after 28 days,^3^ in this research, the mice were carried out up to 15 days post-skin lesion excision, noticing that they did not reach a complete healing process. Moreover, in contrast to our results, where no statistical significance was reported in the percentage of skin lesions along the time of the wound healing process between the ISS and PSCs groups, Ma *et al.* reported statistical significance in the healing rate between the control group and the group treated with MSCs. However, on day 14, Ma *et al.* reported that the healing rate of the MSCs group was around 60%, which was quite similar to the results obtained in this research, where on day 15, the percentage of skin lesions was around 40% in both groups. In addition, it is essential to mention that two days post-skin lesion excision, the injury considerably diminished from 99.46 ± 4.07 and 99.99 ± 4.71 mm^2^ in the ISS and PSCs groups, respectively, to 85.44 ± 3.34 and 86.64 ± 3.96 mm^2^, probably due to rodents' skin that has a panniculus *carnosus* layer, which produces rapid wound contraction following injury.^21^

As previously mentioned, the wound healing process consists of four phases: (1) hemostasis, (2) inflammation, (3) proliferation, and (4) remodeling.^9–11^ According to this, in the histological analysis of this research, we could observe at least three phases of the wound healing process; however, critical histological differences were detected between groups. For example, on day 7 post-skin lesion excision in the ISS group, a hematic scab evidenced the hemostasis phase and the inflammation phase by separating the papillary dermis and migration of inflammatory cells. In addition, some disorganized collagen fibers were observed. In contrast, the hemostasis phase was characterized by a less solid scab in the PSCs group. However, it is essential to mention that the inflammatory response was regulated once a separation of the papillary dermis was not observed in the inflammatory phase, even though the migration of inflammatory cells was evidenced.

Moreover, organized collagen fibers were observed, and the results that agree with Gal *et al.* (2008), who established in diabetic rats an excisional skin wound healing model employing corticosteroid as treatment. In this research, by day six after the surgery lesion, they found a new layer of epithelial cells in the control group, lightly infiltrated with PMN, and a newly created granulation tissue at the bottom of the wounds.^24^

On day 15 post-skin lesion excision (end of proliferation phase) in the ISS group, the scab remained persisting a slight edema sign in the dermis layer with a demarcation line comprised of inflammatory cells, which means that the inflammatory phase was in its final phase. However, granulation tissue, including a smaller amount of collagen, was observed, and an epithelialization was below the covering surface where the scab was placed. In contrast, the PSCs group exhibited a thinner epidermal layer than the ISS group; granulation tissue and new vessels were also evidenced. The proliferation and migration of fibroblasts were decelerated, and the amount of collagen increased with a good deposition and orientation, highlighting the presence of hair follicles. This was partially consistent with Gal *et al.*, who reported that the epidermis regeneration was finished on day 14 of post-surgery in the control group. In addition, the number of fibroblasts and endothelial cells decreased, and the amount of collagen increased simultaneously, forming newly organized fibrils.^24^

As previously mentioned, the ISS (control group) did not exhibit hair follicles, which has also been reported by Nguyen *et al.*, who developed a mouse model of diabetes employing STZ, generating wounds of 6-mm by biopsy punch, declaring that no anagen hair follicles were evidenced in the wound healing process, stating that some factors may influence diabetic wound healing, such as abnormalities in hair follicles resulting in defective hair development and cycling, thereby representing a sign of vascular impairment and organ damage in diabetic patients.^7^

The amount of granulation tissue, early collagen, inflammatory infiltrate, vertical orientation of reticular collagen, and minimum amount of mature collagen are the symbol of delayed healing.^25^ In addition, it is essential to mention that collagen type III is secreted at the early stages of the remodeling phase; it appears between 48 and 72 h and is maximum between five and seven days. After a year or more, the dermis gradually returns to its preinjury phenotype, with a predominance of collagen type I.^11^ The histopathological analysis through picrosirius red stain allowed us to analyze the remodeling phase in the granulation tissue, evidencing that type III collagen fibers are more abundant in the PSCs group compared to the ISS group. Furthermore, the content of these fibers increased over time, which means that the PSCs group was crossing in the remodeling phase since day 7 post-skin lesion excision, while the ISS group reached this phase until day 15 post-skin lesion excision.

Considering the aforementioned analysis, the general histologic picture of healing in the PSCs group was enhanced compared to the ISS group.

Among the main sources of cells that might be used for wound healing and regeneration of injured skin are embryonic stem cells, induced pluripotent stem cells, and adult stem cells. The reported results using embryonic stem cells or PSCs evidenced epidermis, hair follicles, sebaceous gland regeneration, reduced scar widths, and the promotion of collagen maturity.^26^ Indeed, some studies have already reported the use of stem cells in wound healing in diabetes, such as Barcelos *et al.*, who evaluated the healing potential of the human fetal aorta-derived CD133^+^ progenitor cells in a model of ischemic diabetic ulcer, reporting an accelerated wound closure.^27^

Furthermore, as mentioned in the Results sections, the epidermis thickness was evaluated. As observed, the thickness of the epidermis increased throughout the healing time, indicating that even though no statistical significance was found, the ISS group presented a thicker epidermis than the PSCs group at day 7 post-skin lesion excision. However, on day 15, the PSCs group exhibited a thicker epidermis than the ISS group. Regarding this, it is known that the thickness of the epidermis depends on re-epithelialization events, which are controlled and accelerated by the regulation of inflammatory molecules induced by stem cells.^28^ Xiao *et al.* have mentioned that some studies that have evaluated the use of stem cells in wound healing have been shown to promote the re-epithelialization of skin wounds by inducing the proliferation and differentiation of keratinocytes. In addition, they also reported a decreased inflammatory cell and proinflammatory cytokines, which accelerates the wound healing process by regulating the size of the thickness of the epidermis, generating a controlled process.^29^ In this sense, it explains why in the initial stages of wound healing, especially in the inflammation phase (7 days), the thickness of the epidermis of the PSCs group is reduced compared to the ISS group. However, as mentioned earlier, in this research, we could only evaluate the initial phases of the wound healing process, so assessing the final stage of the proliferation phases would require several weeks.

In this study, FT-IR spectroscopy was used to detect biochemical changes in the wound healing process, the indirect amount of some molecules involved, and the different wound healing stages. Even though this research was conducted in mice, our results agree with the ones reported by Lucassen *et al.*,^30^ Tang *et al.*,^31^ and Ali *et al.*^32^ They carried out different experiments, including FTIR analysis in human skin, reporting bands related to amide I, amide II, lipids, ceramides, and collagen. In the same way, our results agree with Ghimire *et al.*^33^ and Castro *et al.*,^34^ who directed their studies on skin mice and rats, respectively, obtaining similar spectra to the ones reported in this study.

Castro *et al.* reported a higher content of carbohydrates, proteins, lipids, and nucleic acids after burn trauma in rats, demonstrating an intense metabolic activity associated with the cascade of events triggered by the thermal injury, indicating the activation of biological events related to homeostasis after the trauma, highlighting that according to the wound healing process progressed the metabolic activity decreased;^34^ which is consistent with the observed in this study, where proteins [amide I (1666 cm^−1^) and amide II (1549 cm^−1^)], lipids (1737 and 1456 cm^−1^), and nucleic acids (1085 cm^−1^) exhibited higher absorbance in the dermis of the PSCs group at day 7. After that, it decreased on day 15, highlighting that on day 7 post-skin lesion excision, the PSCs group exhibited higher absorbance of these bands than the ISS group, but on day 15, the ISS group, the band related to proteins and lipids at 1456 cm^−1^ showed higher absorbance than the PSCs group, indicating that the ISS group starts with this metabolic activity until day 15. This agrees with the histological results of the ISS group's delayed wound healing process.

Moreover, in the epidermis section, the bands related to lipids and nucleic acids on day 7 in the PSCS group presented a higher absorbance than the ISS group, and on day 15, post-skin lesion excision, these bands started to decrease, but the bands attributed to proteins increased. Therefore, analyzing the spectra, we can say that the PSCs group presented a more significant metabolic activity than the ISS group; moreover, the dermis section exhibited greater metabolic activity than the epidermis section, probably because the dermis is thicker than the epidermis and due to the granulation process, which is carried out in the dermis.

In addition to the above discussion, it is essential to examine the participation of lipids in the wound healing process once the band associated with lipids (1737 cm^−1^) exhibited a higher absorbance in the PSCs group compared to the ISS group on days 7 and 15 in both sections (epidermis and dermis). Moreover, the band at 1456 cm^−1^ also showed a higher absorbance in the PSCs group on day 7. About this, Castro *et al.* have declared that the higher amount of lipids involves its participation in cell signaling (such as phosphatidic acid, phosphoinositide, and diacylglycerol) as well as in energy sources (triglycerides, diglycerides, cholesterol, free fatty acids), which is required for cell proliferation, migration, and cellular matrix synthesis.^34^ Moreover, Silva *et al.* have stated that fatty acids interfere with the maturation and differentiation of the stratum corneum and inhibit the production of proinflammatory eicosanoids, reactive species, and cytokines, thus influencing the inflammatory response and possibly wound healing,^35^ which is also consistent with the histopathological results of this study, once the inflammatory response was regulated in the PSCs group. Furthermore, paying attention to the wound healing stages, we could stage that the PSCs were the inflammatory stage according to the biochemical characteristics presented in the FTIR analysis on day 7.

It is known that skin wounds heal by first intention or granulation; granulation is a complex biological process crucial for wound healing, replacing damaged tissue with living. Granulation tissue grows on a wound's surface by forming connective tissue and blood vessels. The granulation tissue is predominantly composed of collagen (mainly types I and III) and elastin, which present vibrational modes at 1400, 1340, and 1280 cm^−1^, as well as glycoproteins, proteoglycans, glycosaminoglycans, and hyaluronic acid, which are essentially constituted by carbohydrates (monosaccharides, disaccharides, polysaccharides) and have vibrational modes peaking at 1029 and 1082 cm^−1^. The early granulation tissue contains hyaluronic acid (glycosaminoglycan) and fibronectin. The concentration of hyaluronic acid within the wound area quickly decreases while collagen takes its place.^10,36^

Therefore, the observation of granulation tissue means that the wound is progressing from the inflammatory phase to the proliferative healing phase. The analysis of the spectra in the dermis section evidenced that on day 7 in the PSCs group, the bands associated with collagen and elastin showed a greater absorbance than the band related to monosaccharides, disaccharides, polysaccharides; nevertheless, on day 15 the monosaccharides, disaccharides, and polysaccharides band evidenced a higher absorbance, which means that the synthesis of these molecules began and that the early granulation phase is taking place. Nonetheless, the band associated with the ISS group's monosaccharides, disaccharides, and polysaccharides never increased. Moreover, this band in the PSCs group exhibited higher absorbance than the ISS group, confirming the acceleration of wound healing in the PSCs group.

Fibronectin is an adhesive glycoprotein that plays a crucial role in wound healing, particularly in extracellular matrix formation and re-epithelialization. This glycoprotein plays a role in the early phases of wound healing; after wounding, it participates in the homeostasis process by releasing platelets into the blood flow to form a platelet plug; moreover, in the inflammatory phase, fibronectin opsonizes the extracellular matrix debris. Beyond that, it activates macrophages so that they can phagocytize the debris. However, the central role of fibronectin is extracellular matrix formation. First, plasma fibronectin forms a provisional fibrin–fibronectin matrix; the provisional matrix is degraded and replaced by granulation tissue. Finally, when the scar matures, fibronectin is broken down to create a place for collagen deposition, which gives strength to the final scar.^37,38^

The fibronectin polypeptide chain comprises several repeats or modules of Fn1, Fn2, and Fn3. Fn1 modules are composed of two layers of antiparallel β-sheets held together by hydrophobic interactions. Fn2 modules are found together with Fn1 modules; their structure, two double-stranded antiparallel β-sheets connected by loops, suggests that a ligand such as collagen may bind to this module through the interaction of hydrophobic amino acid side chains, the structure of Fn3 is conformed by a sandwich of antiparallel β-sheets with a hydrophobic core.^39^ Considering the above-mentioned discussion, by analyzing the secondary structure of proteins through the second derivative of the FTIR spectra, we attributed the band at 1685 cm^−1^ (β sheets) to fibronectin. Furthermore, these bands exhibited a higher absorbance in the ISS group on days 7 and 15 in the epidermis and dermis sections than in the ISS group, agreeing with the results about immunoregulation on day 7 post-skin lesion excision in the PSCs group. This could explain why the ISS group's early phase of wound healing showed a greater absorbance of the band related to fibronectin once it activates macrophages. However, these bands showed a lower absorbance in the PSCs group, evidencing immunoregulation. In addition, on day 15, this band remained higher in the ISS group, which could be attributed to the formation of the provisional fibrin–fibronectin matrix, and remembering the previous results, on day 15, the PSCs group was in the granulation phase, where the provisional matrix is degraded and is replaced by granulation tissue, the reason by which this band showed a lower absorbance in the PSCs group. In addition, some authors, such as Sanden *et al.*, have declared that a higher content of β-sheet structures may contribute to a stiffer structure,^40^ which could be correlated with the scarring process.

Likewise, the band at 1651 cm^−1^ attributed to α-helices of keratin analyzed in the epidermis sections evidenced a higher absorbance in the PSCs group, which increased at day 15. This is consistent with the above-mentioned discussion; keratin is a protein needed for wound healing and tissue recovery that provides tissue robustness.^39^ Moreover, Beaumier *et al.* have declared that in the proliferation phase, the involved cells are macrophages, pericytes, lymphocytes, angiocytes, neurocytes, fibroblasts, keratinocytes, and epithelial cells. Each cell develops different functions; for example, the “framer” cells are fibroblasts, which secrete the collagen framework on which further dermal regeneration occurs. In addition, specialized fibroblasts are responsible for wound contraction. The “roofer” and “sider” cells are the keratinocytes responsible for epithelialization. In the final stage of epithelialization, contracture occurs as the keratinocytes differentiate to form the protective outer layer or stratum corneum.^41^ Thus, once again, the PSCs group showed signs of more advanced stages of healing than the ISS group.

On the other hand, at 1660 cm^−1^, it can be distinguished the band associated with collagen in the dermis section, a component that has already been discussed; however, we can observe that this band exhibited a higher absorbance in the PSCs group at day 7, remaining in absorbance at day 15; in contrast, this band in the ISS group star to express higher absorbance until day 15.

Moreover, the disordered structure of proteins at 1638 cm^−1^ was also detected, which showed a higher absorbance in the PSCs group on day 7 in the epidermis and dermis sections; nevertheless, the ISS group exhibited an increment at day 15 in both sections. About this, the characteristics of normal scars result from the changes in extracellular matrix structure and composition in the dermis; the most significant difference between normal tissue and scar tissue is the orientation of the fibrous matrix.^42^ Herein, we could detect disordered structures of proteins at early stages (day 7) in the PSCs group, which is consistent with the results obtained in the analysis of other bands.

In the same way, the mapping analysis through FTIR and the biochemical content through the calculation of the areas under the curve reinforced the results presented in the FTIR spectra analysis. The PSCs group exhibited a higher content of lipids at days 7 and 15 post-skin lesion excision, which has been previously discussed, highlighting that the analysis of the lipids content evidenced a greater content in the epidermis region, which is consistent with the skin histology. The epidermis has a very active synthesis of cholesterol, fatty acids, and ceramides; these lipids are produced by keratinocytes and play an essential role in the skin's barrier function. The disruption of the skin's barrier function results in a rapid and marked increase in epidermal cholesterol and fatty acid synthesis,^43^ observed in both groups (ISS and PSCs groups) at day 7 post-skin lesion excision.

Regarding ceramides of amide I and amide II mapping, the ISS group on day 7 presented a greater content of this molecule, probably due to ceramides inducing fibroblast apoptosis, which is a necessary step for the evolution of granulation tissue into scar tissue.^44^ Therefore, the apoptosis step was carried out in the ISS group. However, on day 15, the ceramide content considerably diminished in the ISS group, showing a greater ceramide content in the PSCs group. Tucci *et al.* have reported that there is a relationship between higher ceramide skin content and the improvement in skin hydration, participating in the maintenance of physiological barrier function, controlling its hydration status and counteracting stressing stimuli.^45^ Thus, considering these details, the wounds treated with PSCs exhibit a better physiological function as a barrier at day 15. However, the analysis of the ceramide content evidenced that even though the dermis regions showed a greater content of these molecules in the dermis section on days 7 and 15 post-skin lesion excision, no statistical significance was observed. Nevertheless, on day 15, the ceramide amide II content was higher in the epidermis region in the PSCs group than in the ISS group, reinforcing those mentioned above about the PSCs group exhibiting a better physiological function as a barrier.

The mapping related to collagen content showed that the content of this molecule was greater in the PSCs group on days 7 and 15 post-skin lesion excision. As it is known, collagen is a critical component of the extracellular matrix and plays a critical role in the regulation of the phases of wound healing; in response to injury, collagen induces platelet activation and aggregation, resulting in the deposition of a fibrin clot at the injury site;^46^ this could be the reason for the higher collagen amount at day 7, which decreased at day 15 in both groups. In the same way, the biochemical content of this molecule analyzed through the area under the curve evidenced that the PSCs group showed a higher amount of collagen on days 7 and 15 post-skin lesion excision.

About the collagen triple helix (1660 cm^−1^), it has been reported that the basic structural unit of collagen fibers is tropocollagen (a single collagen triple helix molecule), which can self-assemble to create larger protein structures giving rise to different kinds of collagens. A notable feature of the collagen triple helix is that the amino acids occupying Xaa and Yaa positions are solvent-accessible. Because of this, these residues would be predicted to play essential roles in interactions with other molecules, such as extracellular matrix proteins,^47^ which might be correlated with the proliferation phase of the wound healing process, specifically in the creation of new collagen fibers. As expected, this molecule showed a higher content in the PSCs group on days 7 and 15, which might correlate with the accelerated regeneration process in this group, highlighting that this molecule is almost absent in the ISS group on day 15. The biochemical analysis also observed these results by calculating the areas under the curve.

As previously mentioned, the ISS group showed a greater content of disordered structure of proteins at day 15 than the PSCs group, which has been previously discussed, highlighting that this might be associated with hypertrophic scars once it has been described that the physical properties of hypertrophic scars are a consequence of the disordered organization of the narrower collagen fibrils, which are arranged chaotically.^48^

The collagen fibers aligned mapping analyzed through the amide I/amide II ratio displayed that the ISS group on day 15 exhibited unordered collagen fibers. In the context of the microarchitecture of dermal collagen, Ribeiro *et al.* have declared that collagen fibers form an arrangement allowing the continual movement of the individual fibers engendering the strength of collagen to resist severe stretch; the strength and softness of the skin depend on the supra-organization of their collagen fibers.^49^ Higher values represent an unordered collagen fiber structure with multiple directions; in this sense, on day 7, both groups showed almost the same organization of collagen fibers; nevertheless, on day 15, the PSCs group showed an ordered fiber structure contrasting with the ISS group, reinforcing those mentioned above about the disordered structure of proteins.

Finally, the α–β transition content was evaluated through the calculation of the β-sheet/α-helix content ratio; it has been described that the transition of the α-helices to β-sheets forms a stable wound dressing. Moreover, Litvinov *et al.* have declared that the α-helix to β-strand conversion occurs in the fibrin clot as a part of forced protein unfolding, indicating that forced fibrin elongation and compression are accompanied by a significant α–β conversion under relatively high deformation. Highlighting that the α–β transition in response to stress has been demonstrated for several filamentous proteins, the ability of fibrin to undergo the α–β transition and aggregation may result in the formation of tightly packed β-sheets, fibrin clots displaying amyloid-like features and accumulation of β-amyloid peptide (Aβ), making fibrin clots more resistant to proteolytic degradation.^20^ As expected, the PSCs group showed a higher content of α-β transition on days 7 and 15 post-skin lesion excision than the ISS group.

It is essential to consider that we analyzed the skin of diabetic mice. In addition, some authors, such as Campos de Macedo *et al.*, have stated that the skin of diabetes patients suffers morphological and biochemical alteration due to the synthesis and/or degradation of the intracellular matrix, presenting ultrastructural modifications on fibroblast, collagen, and elastic fibers. In the same way, skin surface lipids are reduced, which is consistent with the data obtained herein, where it is observed that the content of lipids, collagen, and ceramides is decreased in the ISS and PSCs groups compared to the healthy skin.^6^

## CONCLUSIONS

In summary, the wounds treated with PSCs showed a faster wound healing process, less inflammatory cellular infiltration, more ordered structures, and well-arranged collagen fibers. Moreover, the restoration of hair follicles was also observed.

However, it is challenging to contemplate the regeneration of the total skin, considering that the skin harbors three distinct PSCs niches (bulge of the hair follicle, the base of the sebaceous gland, and the basal layer of the interfollicular epidermis),^29^ which could increase the knowledge of the process of the non-healing wound.

In the same way, the biodistribution of the PSCs and inflammatory factors in the skin and other tissues should be studied to have a comprehensive understanding before proposing the use of these cells in clinical protocols.

## METHODS

### Pluripotent stem cell culture

The mouse PSCs (ATCC; SCRC-1011) were seeded at a density of 50 000 cells per cm^2^ on a monolayer of mouse embryonic fibroblasts (MEF), which were previously mitotically inactive for PSC growing. We used embryonic stem cell basal medium (ATCC; SCRR-2010) supplemented with 15% fetal bovine serum, 0.1 mM 2-mercaptoethanol (Invitrogen; 21985023), and 1000 U/ml mouse leukemia inhibitory factor (EMD Millipore; ESG1107). The culture dishes were incubated at 37 °C in a humidified 5% CO_2_ and 95% air incubator. When the cultures in colonies reached 70% confluency, doses of 5 × 10^5^ PSCs were obtained and resuspended in 500 *μ*l of isotonic salt solution (ISS).

### Study groups and diabetic murine model with a skin lesion excision

This experimental work followed the Norma Oficial Mexicana Guide guidelines for using and caring for laboratory animals (NOM-062-ZOO-1999) and the disposal of biological residues (NOM-087-ECOL-1995). The animals employed were males, weighing between 25 and 35 g, and NIH strains of 10 weeks of age. They were kept in metabolic cages (Allentown Inc.; EcoFlo Rack) in humidity (50%–60%) and constant temperature conditions (21 ± 1 °C) with a 12 h light/dark cycle and had free access to a standard diet at all times.

Twenty mice were randomly divided into two groups: the ISS group or control, and the PSCs group or experimental group (n = 10); the control group received ISS as treatment and the experimental group PSCs. Both groups were induced to diabetes, receiving an intraperitoneal (i.p.) injection of 175 mg/kg of streptozotocin (STZ) [2-deoxy-2-(3-methyl-3-nitrosoureido)-D-glucopyranose].^19^ To verify the diabetes condition, capillary glucose was evaluated on days 3, 10, 16, and 21 post-diabetes induction using a glucometer (OneTouch Ultra 2, Johnson & Johnson).

A trichotomy in the interscapular region was done to establish the excisional skin model in diabetic mice six days after diabetes induction; subsequently, a depilatory cream was applied to remove residual hair. Then, under anesthesia (10 mg/kg of ketamine and 1 mg/kg of xylazine) and after aseptic and antiseptic procedures, an excisional lesion of 1 cm^2^ up to the muscle fascia on the dorsal area was made employing a scalpel number 11.

The skin healing process was examined on days 7 and 15 post-skin lesion excision, for the purpose of which five mice of each group were sacrificed over those days. After that, an excision of the scarred skin (biopsy) was made to carry out a morphometric, histological, and Fourier-transform infrared (FTIR) microspectroscopy analysis.

Regarding the administered treatments, as previously mentioned, the control group received 500 *μ*l of ISS sub-scaringly (under the hematic scab), while the experimental group received 5 × 10^5^ PSCs resuspended in 500 *μ*l of ISS at days 2 and 8 post-skin lesion excision.

For the obtention of healthy skin data, three histopathological samples were obtained from three healthy mice.

### Morphometric analysis

The animals were anesthetized and placed under a pedestal of 35 cm tall for a photographic record of the lesions and scarred skin, which was carried out using a 12-pixel camera. The photographic follow-up was done 24 h after skin lesion excision, and after that, every two days until the sacrifice of the animals.

The areas of the lesions and scars were performed employing the software Image-Pro Premier (Version 9.1, Media Cybernetics).

### Histological analysis

Both groups' biopsies of scarred skin were histologically analyzed on days 7 and 15 post-skin lesion excision. First, the skin samples were embedded in Tissue-Tek (Sakura; 4583) and frozen; subsequently, three skin tissue cryosections of 5 *μ*m were obtained from each histopathological sample using a freezing microtome (Ecoshel; ECO-1900). After that, tissue cryosections were fixed in 4% paraformaldehyde for 30 min at room temperature and rinsed with a phosphate-buffered solution (PBS). Afterward, hematoxylin & eosin and picrosirius red stains were performed following the standard methods. Finally, the histological samples were analyzed using a light microscope (Ti-U Eclipse, Nikon) or a polarizing light microscope (Nikon, Tokyo, Japan) and the software Image-Pro Premier 9.1, examining the thickness of the epidermis and the cellularity index in the dermis, as well as the content and arrangement of collagen type I and III fibers. In addition, the content of the fibers was evaluated by measuring the luminescence emitted by the birefringence of collagen fibers I and III.

### Fourier-transform infrared microspectroscopy analysis

To develop the biomolecular analysis through vibrational spectroscopy, three cryosections of 5 *μ*m from each histopathological sample obtained from both groups' scarred skin were mounted on a gold-coated microscope slide with a gold layer thickness of 100 nm (Aldrich; 643246–5EA). After that, the samples were analyzed three times employing a Cassegrain objective of 16× of an FTIR microscope (Jasco; IRT-5200) coupled to an FTIR spectrometer (Jasco; 6600).

The skin samples were focused and dried at room temperature for about 15 min to remove excess water, measuring the spectra until the absorption bands related to water were undetectable. Each spectrum was collected in the mid-infrared range (4000–400 cm^−1^) at a spectral resolution of 4 cm^−1^ with 120 scans.

Jasco Spectra manager software performed the spectral analysis in the biological fingerprint region (1800–800 cm^−1^). First, FTIR absorbance spectra were normalized using a standard normal variate (SNV) normalization employing the Unscrambler X software (version 10.3, Camo). Then, all spectra of each analyzed microstructure (epidermis and dermis) were averaged according to the group they belonged.

The second derivative was calculated to deepen the spectral analysis using the Savitzky–Golay algorithm with 15-point windows and the second polynomial order using the Unscrambler X software. After that, the second derivative spectra were analyzed in deconvoluted absorption bands to determine the individual vibrational modes that contribute to the FTIR signal using the best-fit peak fitting routine of Origin software (version 6.1, OriginLab Corporation). Finally, the normalized spectra and their second derivative were averaged and plotted using the Origin 6.1 software.

### Biochemical mapping

Micro-FTIR images (FTIRI) were collected employing the FTIR microscope fitted with a liquid nitrogen-cooled MCT (mercury, cadmium, and tellurium) detector, coupled to an FTIR spectrometer using a 32× Cassegrain objective. The absorbance spectra were acquired in reflectance mode at a spectral resolution of 4 cm^−1^ with 120 scans coadded. In addition, biochemical images were obtained by automated mapping of the FTIR microscope's multiple points (IQ mapping).

The analyzed biomolecules were lipids (1737 cm^−1^), ceramides C=O amide I (1666 cm^−1^), ceramides N–H/C–N amide II (1549 cm^−1^), and collagen (1400 cm^−1^). In the same way, the collagen fibers aligned were analyzed through the amide I/amide II ratio, where higher values represent an unordered collagen fiber structure having multiple directions. Moreover, employing the second derivative, the collagen triple helix (1660 cm^−1^) and the disordered structure of proteins (1638 cm^−1^) were also analyzed.^20^ The analysis of each spectral band was represented in a two-dimensional image, the reconstruction of which corresponded to the density distribution of each chemical species within the specimen. Each image represents the integrated absorbance of a specific band of the IR spectra for each pixel of the MCT detector; the red and blue colors represent the strong and weak absorption of the infrared beam. Finally, the assays mentioned earlier were developed with the microscope measurement software (Jasco).

### Biochemical content

To discriminate if the above-studied molecules were presented in the epidermis or dermis, micro-FTIR spectra of the epidermis and dermis on days 7 and 15 post-skin lesion excisions were obtained. After that, the areas under the curve of the bands related to lipids, ceramides C=O amide I, ceramides N–H/C–N amide II, collagen, and collagen fibers aligned (amide I/amide II) were calculated. In the same way, employing the second derivative spectra, the area ratio of the collagen triple helix (1660 cm^−1^) was also analyzed. Moreover, the intensity ratio at 1622/1651 cm^−1^ to estimate the determination of the antiparallel β-sheet/α-helix content ratio was also calculated, which is related to the *α*–*β* transition. This parameter clearly depends on the degree of deformation for fibrin elongation and compression.^20^

### Statistical analysis

All data were performed in triplicate, and all experiments were repeated at least three times. First, one-way variance analysis (ANOVA) was performed, followed by Tukey's test to determine any significant differences. The software GraphPad Prism 7.0 (GraphPad) was employed for this purpose. P values of less than 0.05 were considered statistically significant.

## Data Availability

The data that support the findings of this study are available within the article.
